# Novel Ag@Nitrogen-doped Porous Carbon Composite with High Electrochemical Performance as Anode Materials for Lithium-ion Batteries

**DOI:** 10.1007/s40820-017-0131-y

**Published:** 2017-02-18

**Authors:** Yuqing Chen, Jintang Li, Guanghui Yue, Xuetao Luo

**Affiliations:** 1 Fujian Key Laboratory of Advanced Materials, Department of Materials Science and Engineering, College of Materials, Xiamen University, Xiamen, 361005 People’s Republic of China; 2 Department of Materials Science and Engineering, College of Materials, Xiamen University, Xiamen, 361005 People’s Republic of China

**Keywords:** Nitrogen-doped porous carbon, Ag nanoparticles, Synergistic effects, Lithium-ion batteries

## Abstract

A novel Ag@nitrogen-doped porous carbon (Ag-NPC) composite was synthesized via a facile hydrothermal method and applied as an anode material in lithium-ion batteries (LIBs). Using this method, Ag nanoparticles (Ag NPs) were embedded in NPC through thermal decomposition of AgNO_3_ in the pores of NPC. The reversible capacity of Ag-NPC remained at 852 mAh g^−1^ after 200 cycles at a current density of 0.1 A g^−1^, showing its remarkable cycling stability. The enhancement of the electrochemical properties such as cycling performance, reversible capacity and rate performance of Ag-NPC compared to the NPC contributed to the synergistic effects between Ag NPs and NPC.
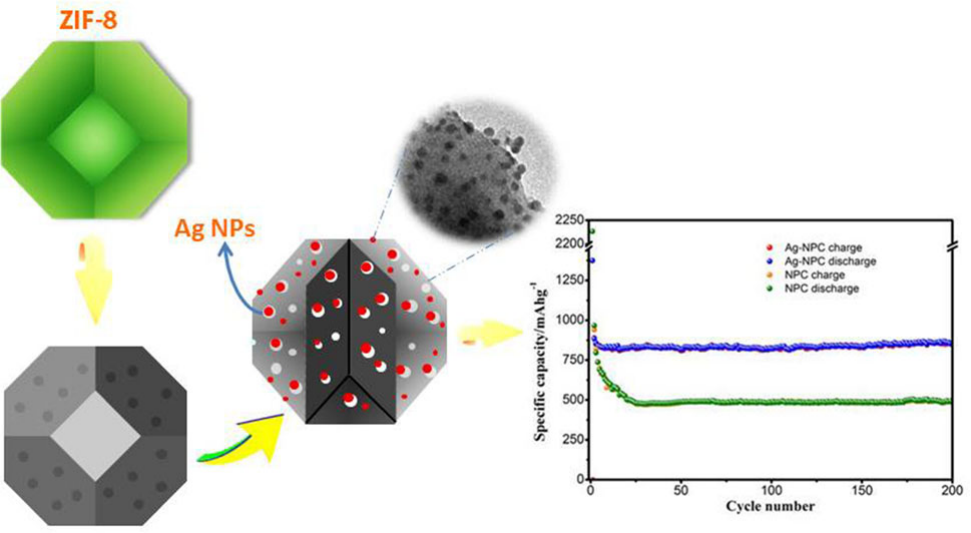

## Highlights

- A novel Ag@nitrogen-doped porous carbon (Ag-NPC) composite was applied to lithium-ion batteries. The encapsulation of Ag nanoparticles (Ag NPs) into NPC boosts reversible capacity from 501.6 to 852 mAh g^−1^.
- Ag-NPC shows a much better cycling performance than NPC due to the synergistic effect of NPC and Ag NPs.

## Introduction

In recent years, lithium-ion batteries (LIBs) have not only been widely used for consumer electronics, but have also proved promising for electric vehicles, owing to their unique advantages, such as high energy and power density, no memory effect and environmental friendliness [1–3]. Currently, graphitic materials are the most commonly used commercial anode materials for LIBs by virtue of their superior cycling stability and high coulombic efficiency [4]. However, due to quite a low theoretical capacity of 372 mAh g^−1^, it would be hard for graphite to meet increasingly high energy requirements in electric vehicles [5]. A variety of materials have been exploited as anode materials for LIBs in the past decades, such as transition-metal oxides [6–8], and silicon-based [9–11] or tin-based [12–14] materials, which have ultra-high theoretical capacity. Unfortunately, these materials react with lithium and are more inclined to form Li_2_O than Li-M alloys. Due to the fact that it is an electrochemically irreversible reaction, it results in a large irreversible capacity [15]. Silver is an appealing option for anode materials, owing to its relatively high specific capacity, which is attributed to the formation of several Ag-Li alloys (up to AgLi_12_) within a very low voltage range (0.25–0 V) [16]. Moreover, silver has the best electrical conductivity among all metals and excellent lithium diffusivity, which can efficiently boost the electrochemical performance [17]. However, silver also suffers from undesirable volumetric expansion during lithium insertion. To alleviate this harmful effect, different strategies have been developed, such as downsizing the particle dimensions to the nanoscale, introducing a porous structure to the solid particles and designing silver-containing composites [18–20]. Carbon is a common matrix for silver. Shilpa et al. used hollow carbon nanofibers as a buffer matrix and embedded silver nanoparticles in them through the coaxial electrospinning method [15]. Hsieh et al. dispersed silver nanorods onto graphene nanosheets by the hydrothermal method [21].

Metal organic frameworks (MOFs) have been attracting increasing attention as carbon sources for anode materials because various types of MOF precursors can result in derived carbon with a uniform, controllable, porous structure and enable innate doping of heteroatoms [22–24]. On the basis of previous research, the nanopores can facilitate rapid electrolyte transfer [25]. In addition, the heteroatom-doped carbon always performs at a higher specific capacity and outstanding cycling stability compared to the non-doped carbon [26–29]. Song et al. prepared a cage-like carbon/nano-Si composite as anode materials by the template method to embed Si nanoparticles into ZIF-8. The resulting nano-Si/C composite showed a higher reversible capacity than many Si/C composites previously reported [30]. Xie et al. fabricated a sandwich-like, graphene-based, porous nitrogen-doped carbon (PNCs@Gr) through the pyrolysis of zeolitic imidazolate framework nanoparticles grown in situ on GO (ZIF-8@GO), which exhibited outstanding electrochemical performance among carbonaceous materials used as anode materials [31].

We used ZIF-8-derived carbon as a matrix for silver nanoparticles (Ag NPs), which can provide not only rigid matrices with nanopores, but also a relatively high nitrogen content. We designed a strategy to incorporate Ag NPs into N-doped porous carbon uniformly via a facile hydrothermal method without any reduction agent. When applied as the anode material for the Li-ion battery, the Ag-NPC showed excellent electrochemical performance over bare NPC, which was attributed to the synergistic effect of Ag NPs and the carbon matrix.

## Experimental

### Chemicals

Methanol (CH_3_OH, Sinopharm Chemical Reagent Co. Ltd, >99.5%), 2-methylimidazole (C_4_H_6_N_2_, Sinopharm Chemical Reagent Co. Ltd., 99%), zinc nitrate (Zn(NO_3_)_2_·6H_2_O, Shanghai Titanchem Co. Ltd., >99.8%), 1-methylimidazole (C_4_H_6_N_2_, Sinopharm Chemical Reagent Co. Ltd., 99%) and silver nitrate (AgNO_3_, Sinopharm Chemical Reagent Co. Ltd., >99.8%) were used. All reagents were used without further purification.

### Preparation of N-doped Porous Carbon (NPC)

ZIF-8 was synthesized according to method reported in the literature [32]. Specifically, a methanolic solution (400 mL) of 2-methylimidazole (6.48 g) and 1-methylimidazole (6.28 mL) was quickly poured into a methanolic solution (400 mL) of Zn(NO_3_)_2_·6H_2_O (5.88 g) and stirred for 2 min and then kept still for 16 h. After that, the solution was centrifuged, washed by methanol and dried at 60 °C for 3 h to produce a white solid (ZIF-8). Then, the solid was ground into powder, followed by heat treatment at 800 °C for 5 h under an argon atmosphere. After letting it cooldown to the room temperature, the obtained product was dispersed into an HCl solution (100 mL, 20 wt% in water) and stirred for 24 h to remove residual metallic Zn and/or ZnO. The mixture was then washed thoroughly with distilled water several times until all the zinc ions were removed. Finally, the resultant product was dried at 60 °C in vacuum oven for 6 h to obtain an N-doped hierarchically porous carbon.

### Synthesis of Ag-NPC Composite

The Ag-NPC composite was synthesized by a facile hydrothermal process without a reduction agent [33]. The NPC (100 mg) was quickly dispersed into an aqueous solution of AgNO_3_ (25 mL, 20 mmol L^−1^), and the mixture was subsequently homogenized by intensely stirring it for 1 h in the dark. Then, the aqueous suspension was heated at 100 °C while gently stirring it under argon flow for 10 min, followed by quickly cooling it with running water. Next, the mixture was centrifuged and washed with distilled water several times and then dried at 60 °C in a vacuum oven for 5 h.

### Characterization

Powder X-ray diffraction (XRD) analysis was performed using a Bruker-Axs D8 Advance X-ray diffractometer with Cu Ka radiation (*λ*=0.15406 nm). The morphology of the sample was studied using a Hitachi SU70 field emission scanning electron microscope (SEM) at 10 kV. The high-resolution transmission electron microscopy (HRTEM) characterization was performed on a Tecnai F30 microscope at an accelerating voltage of 300 kV. The specific surface area and pore size distribution were analyzed by using a TriStar II 3020. The thermogravimetric analysis (TGA) was carried out on a SDTQ600 thermoanalyzer in air. The elemental analysis was performed using a Vario ELIII. The X-ray photoelectron spectroscopy (XPS) was performed using a Thermo Scientific ESCALAB 250Xi with Al *K*α radiation (*hν* = 1486.8 eV).

### Electrochemical Measurements

The active materials, NPC or Ag-NPC composite (70 wt%), acetylene black (20 wt%) and poly(vinyl difluoride) (PVDF, 10 wt%) were diffused in 1-methyl-2-pyrrolidinone (NMP) and stirred intensely to form a homogeneous slurry. Then, a copper foil was coated with the slurry and dried in a vacuum oven at 80 °C for 12 h. The lithium foil was used as the counter electrode as well as the reference electrode. A polypropylene membrane and LiPF_6_ were used as the separator and electrolyte, respectively. The galvanostatic discharge/charge experiments were performed on a Neware battery tester. Cyclic voltammetry (CV) and electrochemical impedance spectroscopy (EIS) measurements were carried out on an Autolab electrochemical workstation (NOVA 1.9).

## Results and Discussion

**Fig. 1:**
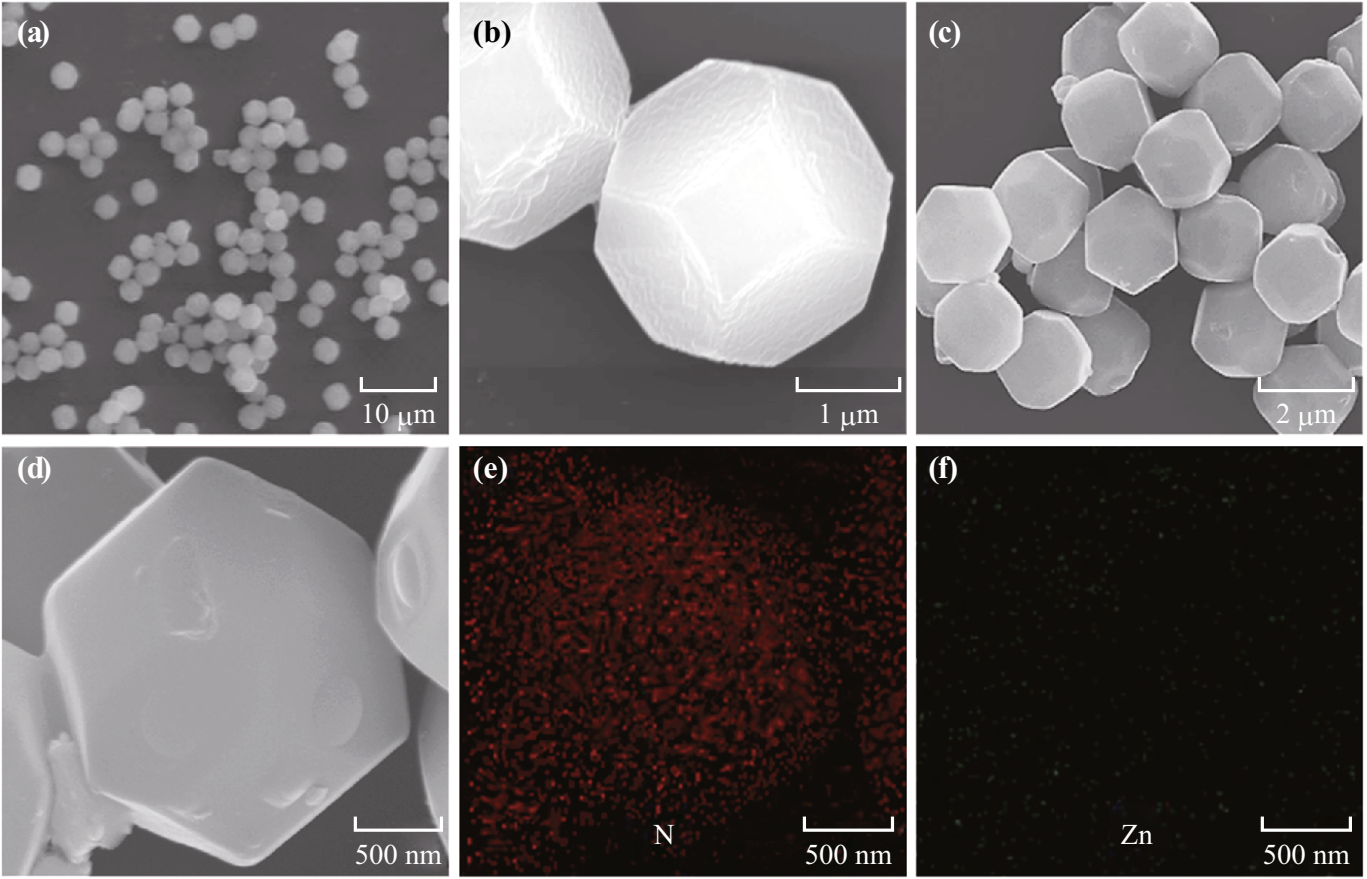
SEM images of **a, b** ZIF-8, **c, d** NPC and **e, f** elemental mapping of NPC

The morphology of the ZIF-8 and derived NPC was detected using a SEM. The ZIF-8 precursor showed an average particle size of about 2 μm (Fig. 1a) and its typical rhombic dodecahedron morphology (Fig. 1b). Notably, after direct carbonization at 800 °C for 5 h in argon flow, the NPC well inherited the morphology of the ZIF-8 precursor with no evident structure collapse (Fig. 1c). And the surface of NPC appears smoother than that of ZIF-8 precursor (Fig. 1d). The elemental mapping provided evidence of the presence of N (Fig. 1e), and the nitrogen content was about 17 wt%, according to the elemental analysis. The elemental mapping of Zn (Fig. 1f) showed that zinc or zinc oxide species were completely removed after acid pickling.

**Fig. 2:**
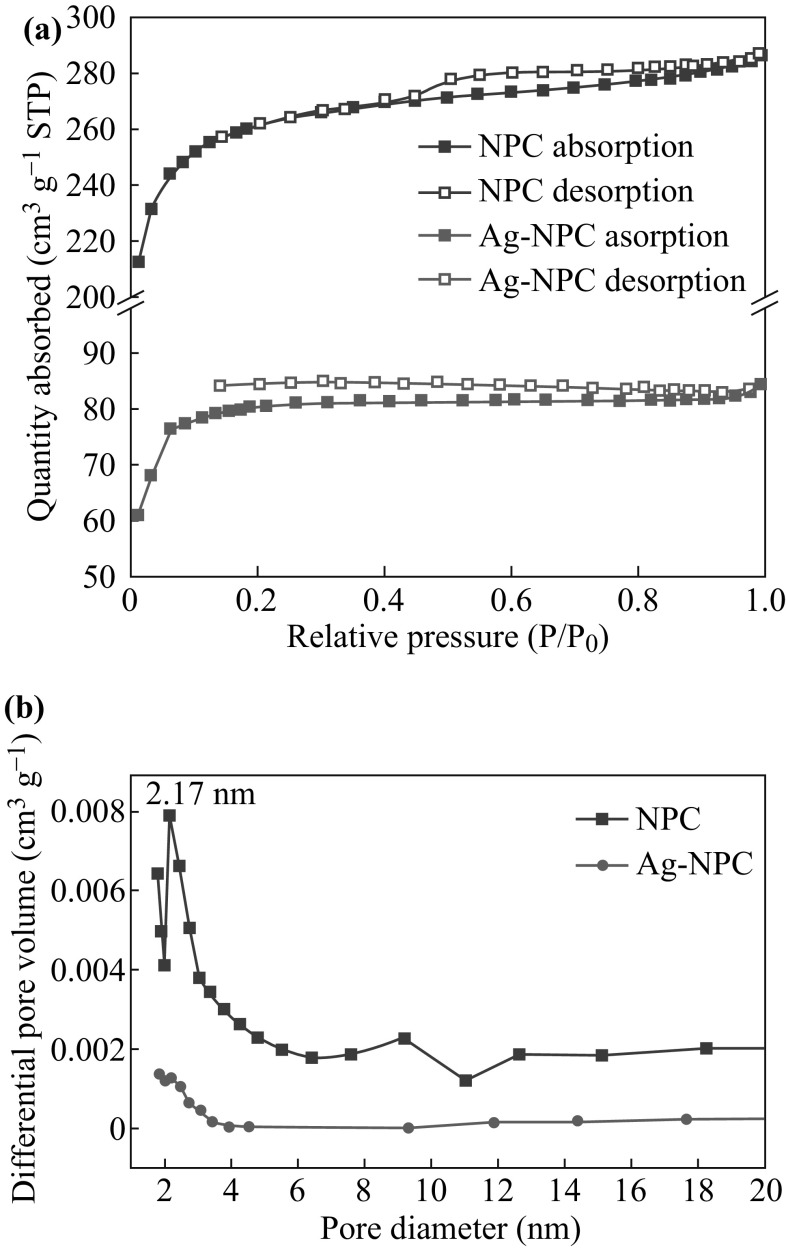
**a** N_2_ absorption–desorption isotherms of NPC and Ag-NPC, and **b** pore size distribution of NPC and Ag-NPC

To get insight into the pore structure evolution before and after the incorporation of Ag NPs, the nitrogen absorption–desorption of NPC and Ag-NPC was measured. The Brunauer–Emmett–Teller (BET) surface of NPC and Ag-NPC was 844.715 and 270.174 cm^3^ g^−1^, respectively. Such a dramatic decrease could be the result of the encapsulation of Ag NPs into hierarchical pores of NPC. The N_2_ sorption isotherm of NPC (Fig. 2a) showed a pseudo-type IV isotherm and a hysteresis loop at a relative pressure (P/P_0_) of 0.4–0.95, which suggests that it is a mesoporous structure, while the Ag-NPC showed a pseudo-type I isotherm, which is consistent with the features of micropores. This indicated that the mesoporous structure of NPC turned into a microporous structure when Ag NPs were embedded, which can be supported by the pore diameter distribution diagram shown in Fig. 2b. The pore size of NPC was mainly centered at 2.17 nm (mesopores), while micropores were dominant in Ag-NPC. The pore quantities in Ag-NPC were much lower than that in NPC for each size, especially for 2.17 nm.

**Fig. 3:**
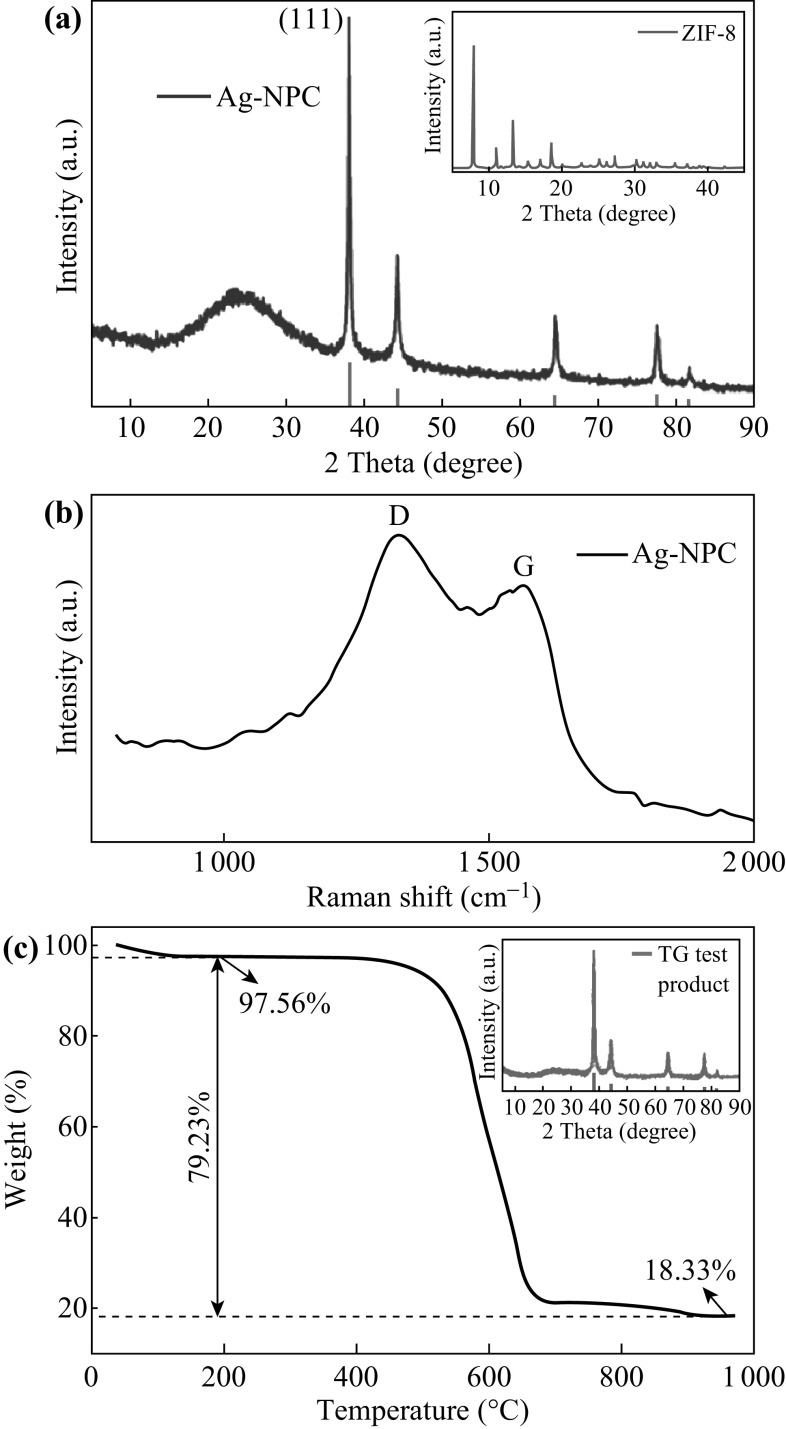
**a** XRD patterns of the Ag-NPC composite and ZIF-8, **b** Raman spectra of Ag-NPC and **c** TGA analysis of Ag-NPC and XRD pattern of the product

Figue 3a shows XRD patterns of in situ synthesized ZIF-8 and Ag-NPC composites. The XRD results of ZIF-8 agreed with the simulated pattern of ZIF-8. The peaks of Ag-NPC at 38.2°, 44.4°, 64.6°, 77.6°, and 81.8° were typical of XRD patterns of metallic Ag. Notably, a weak and broad diffraction peak at 2*θ* with a value of approximately 25° was observed, which was related to the (002) lattice plane of hexagonal graphitic carbon, indicating a low graphitic crystallinity of NPC matrix. This result could be corroborated by Raman spectroscopy of Ag-NPC as well. In Fig. 3b, the D-band represents the disordered graphitic crystallites of carbon and the inner defects of graphitic crystallites, while G-band represents the ordered section. The D/G ratio (*I*_D_/*I*_G_) of Ag-NPC was 1.10, indicating a highly disordered structure of NPC matrix.

To determine the content of Ag NPs, thermogravimetric analysis in air was carried out for the Ag-NPC. As shown in Fig. 3c, a slight weight loss was observed at temperatures lower than 150 °C, which was probably caused by the evaporation of absorbed water. Then, the curve was flat until 500 °C, at which the nitrogen-doped carbon began to decompose. When the temperature increased over 900 °C, the curve became flat again, indicating complete decomposition of the carbon matrix. The resulting product of TGA proved to be metallic silver, confirmed by means of XRD. That is, the weight percentage of Ag NPs in Ag-NPC was 18.79%.

**Fig. 4:**
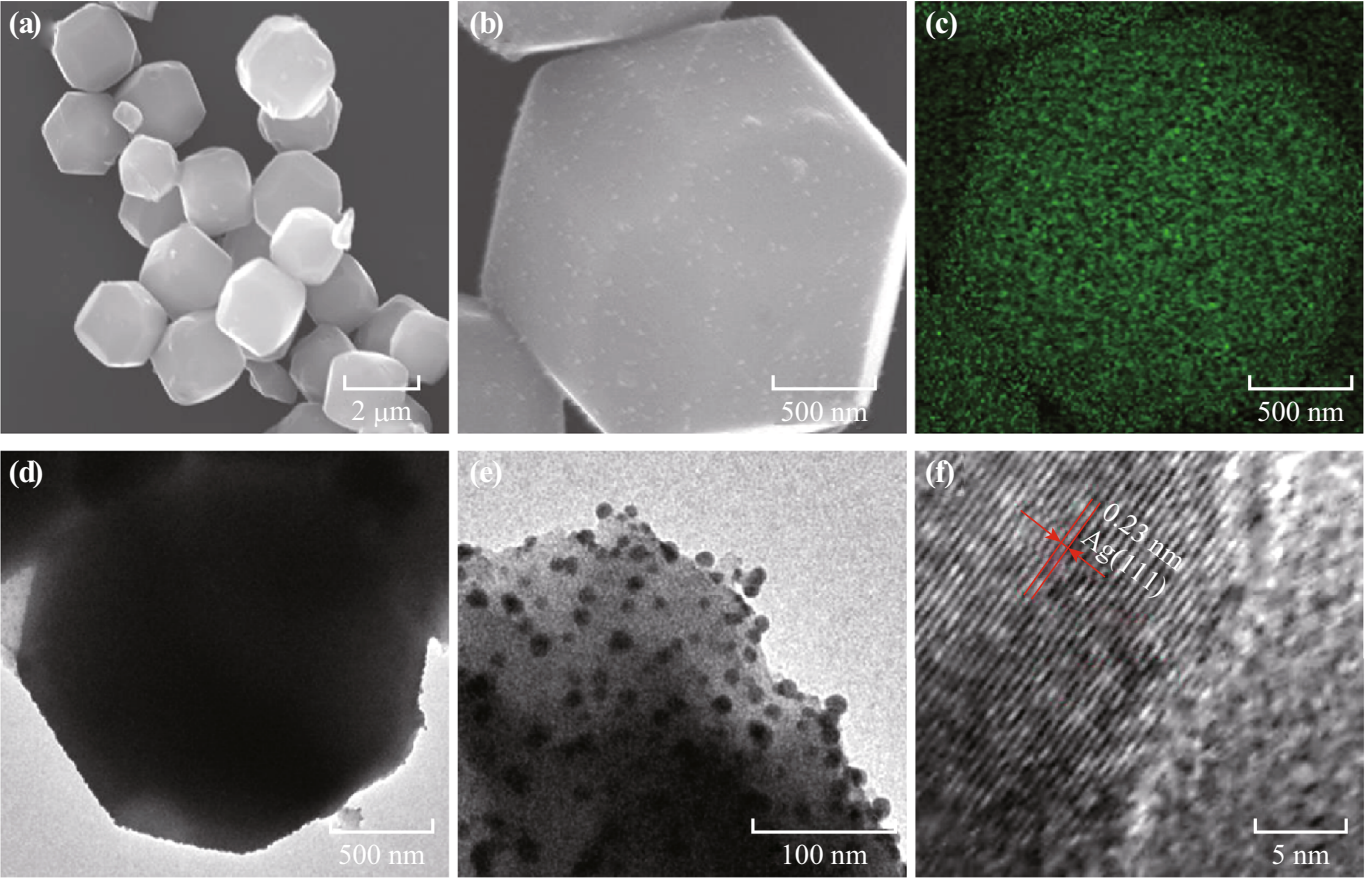
**a, b** SEM micrograph of Ag-NPC, **c** elemental mapping of Ag-NPC, **d, e** TEM image of Ag-NPC and **f** HRTEM of Ag NPs on the surface of NPC

The morphology of Ag-NPC was determined from SEM images. As shown in Fig. 4a, Ag-NPC kept the size and morphology of NPC particles. From the high-magnified SEM image shown in Fig. 4b, it was clear that many nanoparticles were attached to the surface of NPC, which was different from the smooth surface of NPC (Fig. 1d). The elemental mapping in Fig. 4c implied that Ag NPs were uniformly encapsulated in NPC and evenly attached to its surface. A more detailed investigation was performed using HRTEM (Fig. 4d). The particle size of Ag NPs in Fig. 4e ranged from 8 to 20 nm. The lattice distance in the related high-resolution TEM image was about 0.23 nm (Fig. 4f), which was consistent with the interplanar distance of d (111) in the Ag crystal.

**Fig. 5:**
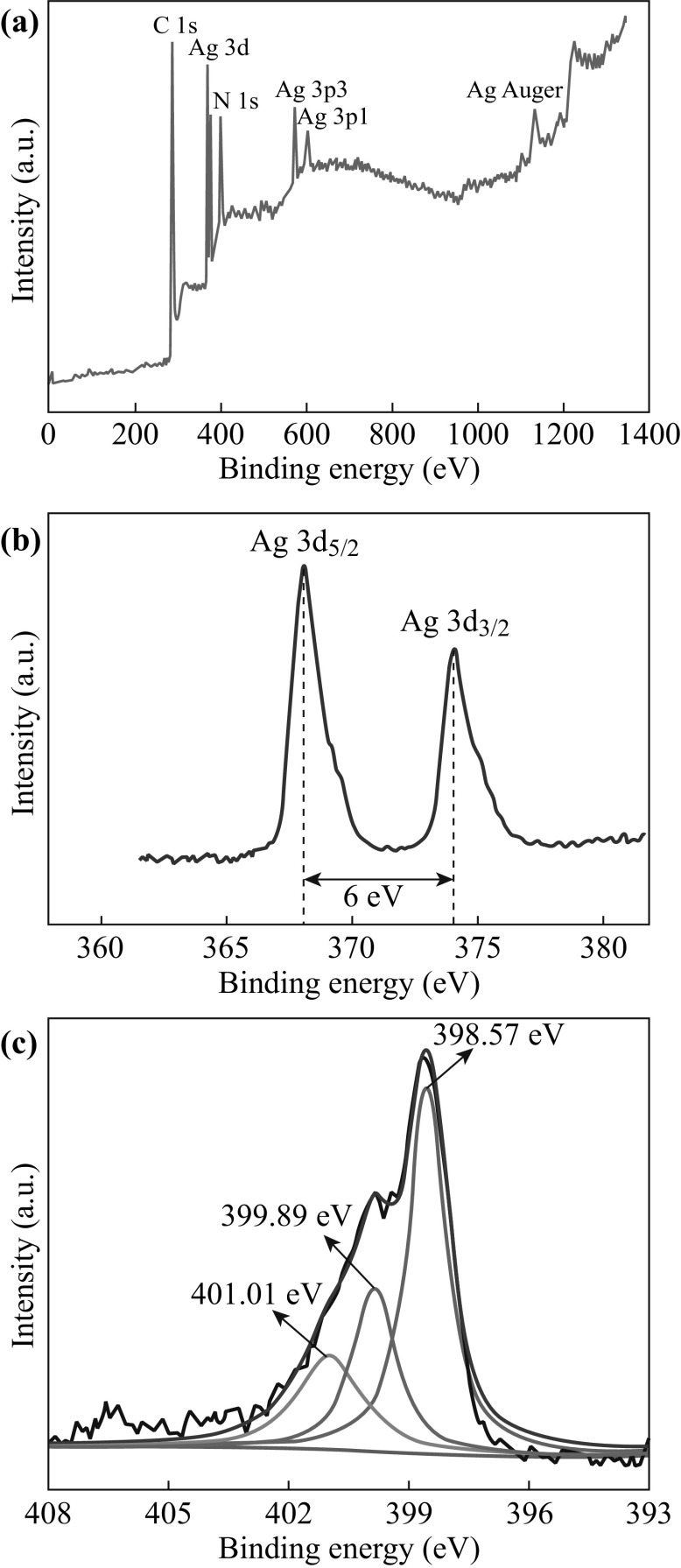
**a** XPS full spectra of Ag-NPC composite. The high-resolution XPS spectra of **b** Ag 3d and **c** N 1s

The chemical nature of the Ag-NPC can be analyzed by XPS characterization, as shown in Fig. 5a. The wide-survey scan XPS spectrum corroborated the presence of C, N and Ag elements in Ag-NPC. The high-resolution Ag 3d spectrum (Fig. 5b) showed the core level binding energies at 368.3 and 374.3 eV that corresponded to Ag 3d_5/2_ and Ag 3d_3/2_, respectively. The spin energy separation of 6.0 eV showed evidence of the metallic nature of silver nanocrystals [34]. The N 1s spectrum (Fig. 5c) can be decomposed into peaks that matched different chemical states, pyridinic N (398.57 eV), pyrrolic N (399.89 eV) and quaternary N (401.01 eV) of nitrogen. Further quantitative analysis of the N 1s spectrum showed that the majority of N in Ag-NPC existed in the form of pyridinic N (≈53.2 wt%), which has a lone pair of electrons that could facilitate the electron transfer [35].

**Fig. 6:**
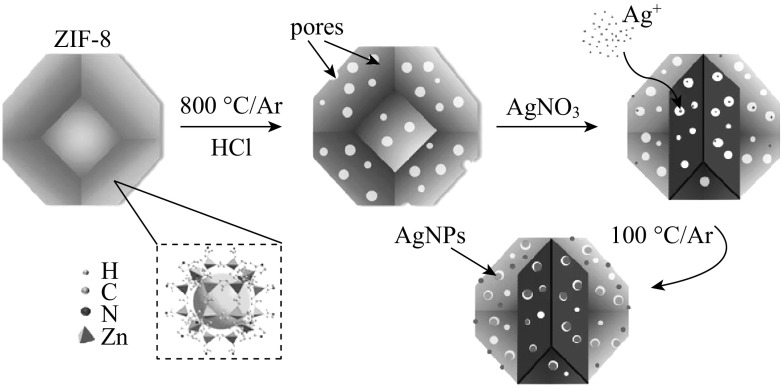
Schematic diagram of Ag-NPC formation process

According to previous discussion and analysis, the schematic diagram of the formation process of Ag-NPC is presented in Fig. 6. ZIF-8 was pyrolyzed at 800 °C followed by acid pickling with HCl to produce N-doped porous carbon. Then, the N-doped porous carbon was immersed into an AgNO_3_ solution and stirred intensely to allow the Ag^+^ to diffuse into the pores inside the NPC and tightly adhere to the pore surface due to electrostatic interaction. Through heat treatment at 100 °C under the argon flow, AgNO_3_ was decomposed into metallic silver. Thus, Ag NPs were uniformly embedded inside the carbon matrix and deposited onto the outer surface of the NPC microparticles.

**Fig. 7:**
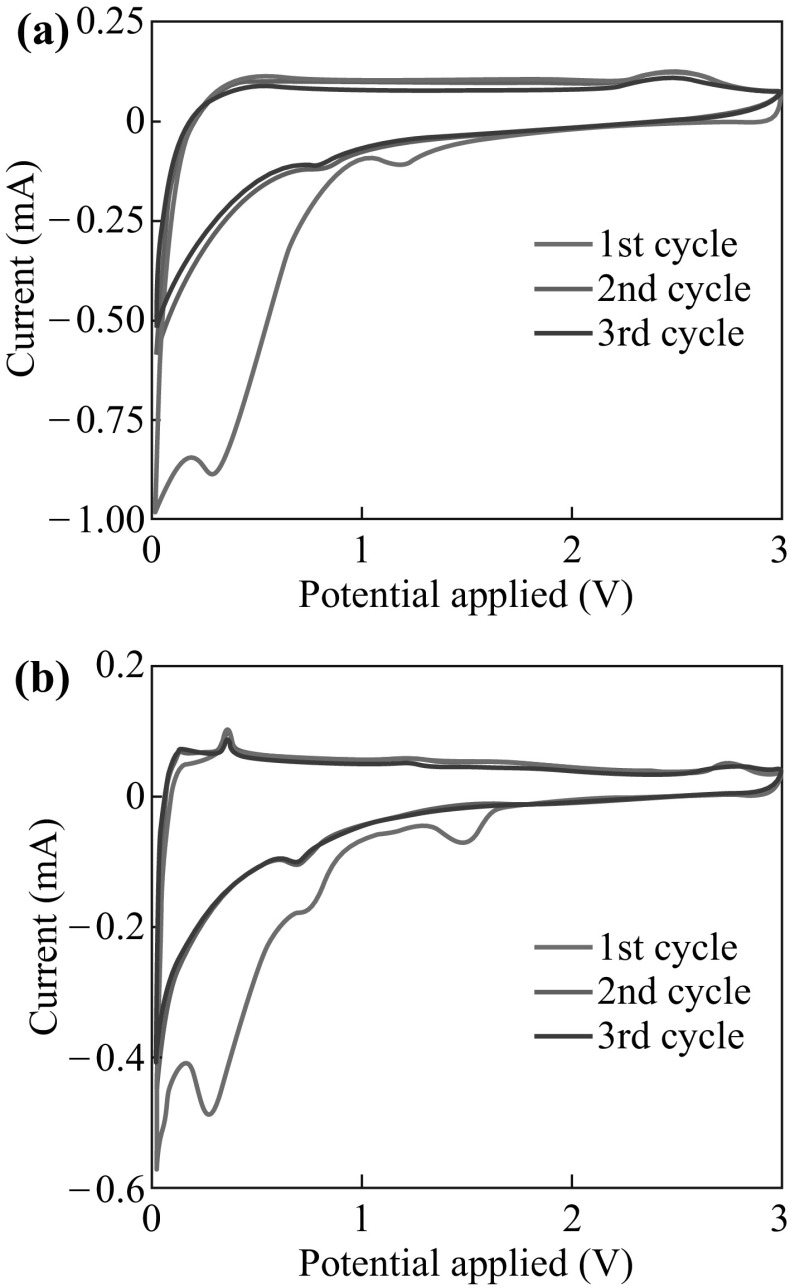
Cyclic voltammogram of **a** NPC and **b** Ag-NPC electrode obtained at 0.1 mV s^−1^ in the potential window 0.01–3 V

The electrochemical properties of the Ag-NPC and NPC were measured. To investigate the electrode reactions of NPC and Ag-NPC during the Li^+^ insertion/extraction process, CV characterization was performed at a scan rate of 0.1 mV s^−1^ between 0.01 and 3 V. Figure 7a shows the CV profile of the NPC. In the first cycle, a broad cathodic peak was observed at 0.2–1.0 V, which was assigned to the formation of the solid electrolyte interface (SEI) films. The sharp peak that appeared near 0 V resulted from the Li^+^ insertion into the NPC. In addition, a relatively weak cathodic peak from 1.0 V to 1.4 V could be due to Li^+^ binding with N atoms on the surface of its internal pores [35, 36]. Notably, for the CV profile of the Ag-NPC (Fig. 7b), the redox peak related to the formation of Li_x_N appeared between 0.7 and 0.9 V, showing a small shift compared to that of the NPC. This phenomenon was because the reaction of Li^+^ with nitrogen was catalyzed by the Ag NPs [37]. Furthermore, a new peak appeared at 1.3–1.7 V after the embedding of Ag NPs. This resulted from the alloying reaction of Li^+^ with Ag NPs. Correspondingly, in the anodic scan, the peaks related to the dealloying process of Li-Ag can be observed at 0.35 and 0.12 V. The electrochemical reaction was described as follows [38],

$$x{\text{Li}}^{ + } + x{\text{e}}^{ - } + {\text{ Ag}} \leftrightarrow  {\text{Li}}_{x} {\text{Ag}}$$

We found that the third cycle almost overlapped with the second cycle in the CV profile of Ag-NPC in contrast to NPC, proving that the Ag-NPC displayed a better cycling stability than the NPC did.

**Fig. 8:**
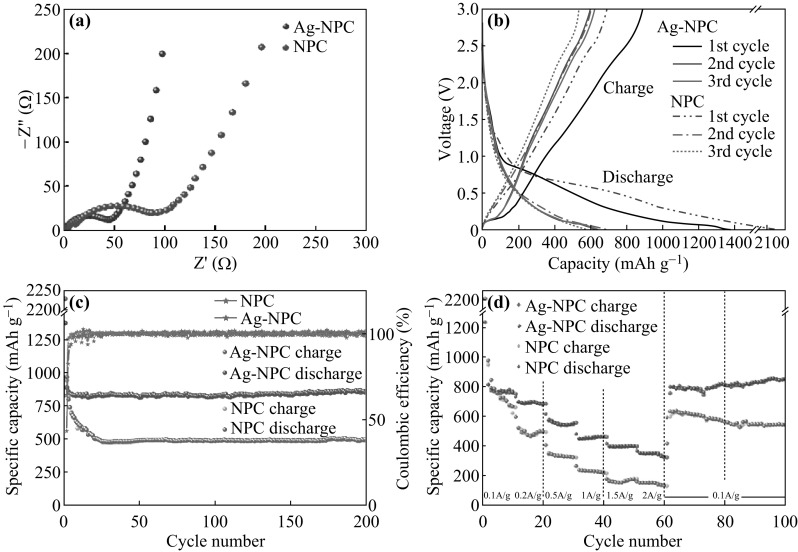
**a** Electrochemical impedance spectra, **b** the galvanostatic discharge–charge profiles at 100 mA g^−1^, and **c** the cycling properties and **d** rate capabilities of NPC and Ag-NPC

Figure 8a shows the analysis of electrochemical impedance spectroscopy (EIS) measurement of NPC and Ag-NPC. Each Nyquist plot showed a semicircle in the high-to-medium frequency region, which was related to the charge-transfer process [39]. The charge-transfer resistance of the Ag-NPC electrode was 50 Ω, much smaller than that of the NPC electrode (100 Ω). This demonstrated that the encapsulation of Ag NPs efficiently enhanced the charge-transfer process due to improvement in the electrical conductivity. The linear tails in the Warburg region corresponded to the mass transfer of Li^+^ inside the anode material. The Ag-NPC exhibited a larger slope than the bare NPC, indicating a higher Li^+^ migration velocity with the incorporation of Ag NPs. Therefore, it can be concluded that Ag NPs can promote both electron transfer and lithium-ion transfer, which has a significant impact on electrochemical performance [40].

Figure 8b displays the galvanostatic charge–discharge profile of NPC and Ag-NPC for first, second and third cycles at a current density of 100 mA g^−1^. In the initial discharge curve of NPC, a broad plateau appeared near 0.75 V, which was usually related to the formation of the SEI films. The plateau for the Ag-NPC around 0.75 V was much narrower than that for the NPC, signaling that smaller areas of SEI films were formed, resulting in less capacity loss. However, no plateau could be detected in the charge curves of the NPC, implying continuous Li^+^ extraction from the NPC. It was noteworthy that for the charge–discharge profile of the Ag-NPC, several small plateaus can be observed apart from the one at 0.75 V. In the discharge curve, plateaus were at 0.076, 0.10, and 0.2 V, respectively. The charge curve presented several small plateaus within the range of 0.1–0.3 V. The step-like curves correlated with the complex alloying and dealloying processes of Ag NPs with Li^+^.

The cycling performance of NPC and Ag-NPC was investigated at a current density of 0.1 A g^−1^ (Fig. 8c). The initial discharge capacity for NPC was very high (2226.64 mAh g^−1^), but a dramatic capacity loss occurred in the first 25 cycles, leading to a low initial coulombic efficiency (43.41%) as well as poor cycling performance. After the encapsulation of Ag NPs, the initial discharge capacity dropped to 1374.64 mAh g^−1^ though its initial coulombic efficiency improved to 64.33%. In the following cycles, the coulombic efficiency quickly increased above 95%, which meant a better cycling performance of Ag-NPC than NPC. The reason was that Ag-NPC had a much smaller specific surface area than NPC (270.174 vs 844.715 cm^3^ g^−1^), so smaller areas of SEI films were formed during cycles. Because the formation of SEI films contributed significantly to the initial capacity [4], the NPC with a much larger area showed a very high initial discharge capacity. However, the reaction of the formation of SEI was always irreversible, so NPC suffered a dramatic capacity loss in the second cycle. In addition, Ag NPs could improve the quality of SEI films, leading to a better cycling performance. Further, after 200 cycles, the reversible capacity for Ag-NPC remained at 852 mAh g^−1^, which was 96.4% of the discharge capacity in the second cycle, while that for NPC was only 501.6 mAh g^−1^. In other words, the Ag NPs can not only efficiently improve the reversible discharge capacity, but also enhance the cycling stability of the NPC. This remarkable improvement was attributed to the synergistic effect of Ag NPs and the carbon matrix. The theoretical capacity of metallic Ag was 993 mAh g^−1^ (for Li_4_Ag), although it suffered from severe volume expansion and agglomeration, leading to poor cycling performance. In the Ag-NPC, the NPC acted as a robust matrix for Ag NPs, hence, alleviating the volumetric expansion and preventing particles from aggregating. Moreover, the encapsulation of Ag NPs reduced the surface contact of the NPC with the electrolytes, preventing the formation of unstable SEI films and improving the quality of SEI films and thus enhancing the cycling performance [9]. Further, the excellent electrical conductivity of Ag NPs accelerated electron transfer and lithium-ion transfer, thus boosting the electrochemical performance.

It is noteworthy that the calculated theoretical capacity for Ag-NPC was found to be 488 mAh g^−1^ based on AgLi_4_ and C_6_Li, which was much lower than the measured capacity of 852 mAh g^−1^. This phenomenon had also been reported by previous papers [41–43]. This was contributed to the highly disordered structure of carbon matrices, high nitrogen content, as well as the improved conductivity of Ag NPs. The carbon matrix was highly disordered according to the broad peak in the XRD pattern and the Raman spectroscopy results of Ag-NPC discussed above. The defects in the carbon matrix promoted Li^+^ diffusion and intercalation into carbon, thereby improving its reversible capacity [42]. Besides, the high nitrogen content (17 wt%) can efficiently facilitate electron transfer, thus increasing the capacity. Furthermore, the superior conductivity of Ag NPs raised the electrical conductivity of the overall Ag-NPC composite and boosted the reversible capacity.

The rate performance for NPC and Ag-NPC was analyzed at different current rates as shown in Fig. 8d. The reversible capacity for both materials gradually decreased with the current rate. The Ag-NPC delivered a much higher discharge capacity than the NPC did at each current density level. The average discharge capacities of Ag-NPC were 777.69, 696.08, 557.30, 459.47, 397.98, and 344.40 mAh g^−1^ for the increasing current values 0.1, 0.2, 0.5, 1.0, 1.5, and 2.0 A g^−1^, respectively. When the current density was lowered back to 0.1 A g^−1^, its discharge capacity recovered to 851.02 mAh g^−1^ at the 100th cycle. The rate behavior demonstrated its superior rate capacity, which could be attributed to the rigid porous structure of the carbon matrix.

**Fig. 9:**
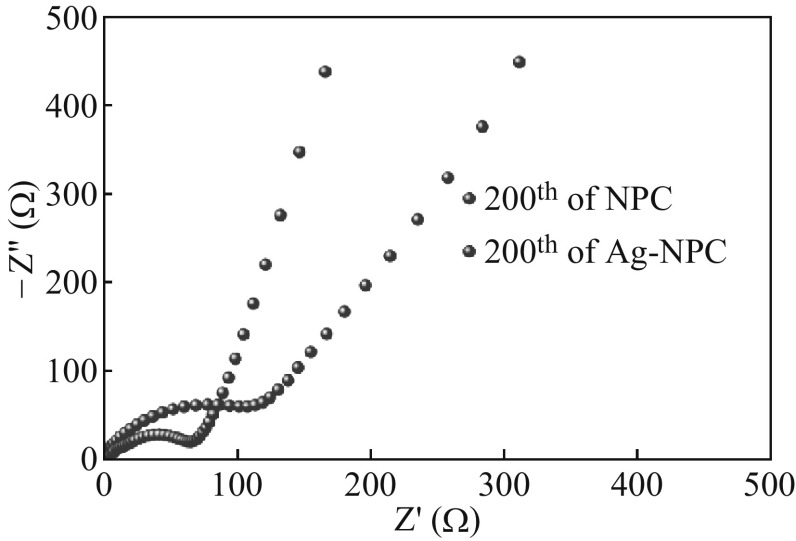
Electrochemical impedance spectra in the 200^th^ cycle of NPC and Ag-NPC

**Fig. 10:**
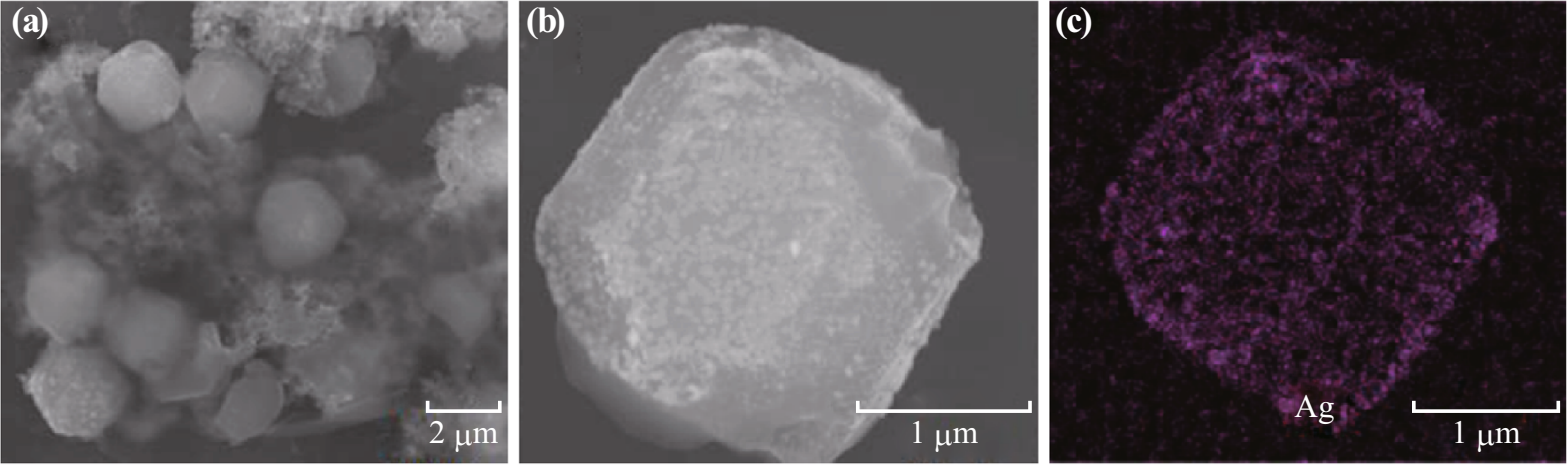
**a, b** SEM micrographs and **c** elemental mapping of Ag-NPC after 200 cycles at 100 mA g^−1^

To further understand the improved cycling performance, we performed EIS measurements after 200 cycles at a current density of 100 mA g^−1^ (Fig. 9). Compared to the EIS results in the first cycle shown in Fig. 8a, both NPC and Ag-NPC displayed a slight increase in charge-transfer resistance after 200 cycles, indicating that they possessed a robust structure, which was mainly attributed to the rigid structure of the carbon matrix that it inherited from the ZIF-8 precursor. The structural stability of the Ag-NPC could be demonstrated visually by SEM. In Fig. 10a, neither agglomeration nor volume expansion to a carbon matrix can be observed. Meanwhile, the original morphology of Ag-NPC, rhombic dodecahedron, was well maintained after 200 cycles at 0.1 A g^−1^ (Fig. 10b).It was noteworthy that Ag NPs were still tightly attached to the surface of the NPC, yet were highly dispersed (Fig. 10c) after 200 cycles, despite the volume expansion of the Ag NPs. This suggests the excellent structural stability of the Ag-NPC composite.

## Conclusions

In summary, a novel Ag-NPC composite with uniformly embedded Ag NPs was prepared through the facile hydrothermal method. The Ag-NPC composite showed remarkable enhancement in the reversible capacity (852 mAh g^−1^ after 200 cycles) as well as cycling performance, compared to NPC without incorporated Ag NPs. This was attributed to the synergistic effect of Ag NPs and N-doped porous carbon. The N-doped porous carbon acted as a robust matrix for Ag NPs, which could alleviate the volumetric expansion and prevent particle aggregation. In return, the Ag NPs that exhibited a relatively high specific capacity as well as superior conductivity could efficiently raise its reversible capacity and enhance cycling performance by improving the quality of SEI films. This research demonstrated that the NPC derived from ZIF-8 was an excellent matrix for nanoparticles in anode materials for lithium-ion batteries. By providing a relatively rigid structure and avoiding particle aggregation, the encapsulated nanoparticles could maximize the reversible capacities.
